# Transparent Conducting Amorphous IZO Thin Films: An Approach to Improve the Transparent Electrode Quality

**DOI:** 10.3390/ma16103740

**Published:** 2023-05-15

**Authors:** Akhmed K. Akhmedov, Aslan Kh. Abduev, Eldar K. Murliev, Victor V. Belyaev, Abil Sh. Asvarov

**Affiliations:** 1 Institute of Physics, Dagestan Research Center of Russian Academy Sciences, Yaragskogo Str., 94, 367015 Makhachkala, Russia; 2 Faculty of Physics and Mathematics, State University of Education, Very Voloshinoi Str. 24, 141014 Mytishchi, Russia; 3 Basic Department of Nanotechnology and Microsystem Technology, Academy of Engineering, RUDN University, 6, Miklukho-Maklay Str., 117898 Moscow, Russia

**Keywords:** TCO, transparent electrode, thin film, multilayer, sputtering, IZO

## Abstract

It is common knowledge that using different oxygen contents in the working gas during sputtering deposition results in fabrication of indium zinc oxide (IZO) films with a wide range of optoelectronic properties. It is also important that high deposition temperature is not required to achieve excellent transparent electrode quality in the IZO films. Modulation of the oxygen content in the working gas during RF sputtering of IZO ceramic targets was used to deposit IZO-based multilayers in which the ultrathin IZO unit layers with high electron mobility (μ-IZO) alternate with ones characterized by high concentration of free electrons (*n*-IZO). As a result of optimizing the thicknesses of each type of unit layer, low-temperature 400 nm thick IZO multilayers with excellent transparent electrode quality, indicated by the low sheet resistance (*R* ≤ 8 Ω/sq.) with high transmittance in the visible range ($\bar{T}$ > 83%) and a very flat multilayer surface, were obtained.

## 1. Introduction

Transparent conductive oxide (TCO) thin films are characterized by high electrical conductivity and high optical transmission in the visible range of the spectrum. A TCO-based transparent electrode (TE) is an integral part for many optoelectronic devices (information display systems, light-emitting diodes, solar cells, etc.) [1,2,3,4]. The unceasing interest in the research of TCO materials is due to the continuous development of the optoelectronics industry in general and the emergence of new types of electronic areas, such as transparent electronics and flexible (and even stretchable) electronics [5,6,7].

Modern trends in the development of the industry lead to the emergence of new, more stringent requirements for both properties of thin-film materials and technological routes for their formation. In particular, for the further wide use of the TCO materials as TEs, it is urgent to find a solution to the “contra-indicated requirement”—the achievement in TCO films of the required specifications of low resistance and high transparency at the lowest possible process temperature [8,9].

All this causes a continuous search for new materials with properties close to those of the “perfect” TE [10,11,12] as well as ways to enhance the functional characteristics of already known TCO materials [13,14,15].

It is known that for TCO-based TEs based on widely used In_2_O_3_, ZnO, SnO_2_ and their compositions, there are performance limits of the TCO films that are determined solely by the electron density in the films. In particular, there is a well-defined limit to the achievable mobility resulting from the electron scattering, regardless of the oxide system or the preparation method used. Experimental data show that this limit is now regularly approached [16,17]. The transparency specification results in an absolute limit to the conductivity of 2.5 × 10^4^ Ω^–1^·cm^–1^ [18,19,20].

In 2003, J.J. Robbins and C.A. Wolden substantiated the theoretical possibility of obtaining highly conductive multilayer thin-film structures by alternating two types of oxide unit layer, one of which is characterized by a high concentration of charge carriers and the second by its high carrier mobility [21]. By direct simulation of periodic structures based on real two oxides (ZnO:Al and GaInO_3_), which differ significantly in electron affinity, the authors showed the possibility of achieving multilayer structure conductivity of about 4 × 10^4^ Ω^–1^·cm^–1^, which is significantly higher than the limit value stated for a single-layer structure. It was also pointed out that the conductivity in such multilayer oxide structures is highly sensitive to the thickness of individual sublayers and the quality of interlayer interfaces. Several other studies also predict that creating regions of high and low carrier density in the body of TCO film by some modulation process may improve its electrical performance [22,23,24].

Currently, among numerous well-studied TCO materials an amorphous indium zinc oxide (IZO) system is one of the more promising materials for the TE application due to its high transmittance, good electrical properties, thermal stability, ultrasmooth surface and easy etchability by organic acids [11,25,26,27,28]. In addition, it can be deposited at low substrate temperature using an industry-friendly technique such as magnetron sputtering [25,29].

Since it is known that the conductivity of amorphous IZO films deposited at low substrate temperature is governed mainly by oxygen vacancies, then there clearly is a strong influence on the carrier transport properties of the oxygen partial pressure applied during sputtering deposition of films [30]. It has already been shown that the IZO films deposited in pure Ar have low resistivity, which is mainly due to a high concentration of donor oxygen vacancies [24,26]. However, the oxygen vacancies’ high concentration adversely affects the electron mobility and transparency of IZO films. At the same time, in low-temperature amorphous IZO films deposited in a reactive medium (Ar/O_2_ gas mix as the working gas), quite high values of Hall mobility are achievable, which are even higher than in polycrystalline ITO films deposited at high substrate temperature [26,29]. As a result, these IZO films exhibit fairly good conductivity (despite a reduced concentration of donor oxygen vacancies), as well as high transparency in the visible- and near-IR ranges.

Based on these facts, it seems interesting to investigate how the periodic change in the composition of the working gas during magnetron sputtering deposition affects the functional properties of amorphous transparent conductive IZO films. In other words, it is important to fabricate a multilayer thin-film periodic structure consisting of interleaved donor-rich and high-mobility unit layers based on a single ionic oxide system of In_2_O_3_–ZnO by programmable periodic changes in the oxygen content in the composition of the working gas during RF magnetron sputtering. The concept of spatial separation of free electron supplier layers and high-mobility layers into which free electrons are supplied by the former and transported, engineered on the base of a single material (IZO), is promising, since this multilayer structure would be free from problems associated with multiple defect-rich heterointerfaces, characteristic for the above-mentioned multilayer structures based on two different materials [21,22,31].

## 2. Materials and Methods

Our earlier studies of the influence of technological conditions of RF sputtering deposition on the functional properties of IZO films showed that the optimal substrate temperature for the deposition of amorphous IZO films is 100 °C [29]. Upon sputtering in a pure argon medium, the transport of carriers in the IZO film is characterized by high free electron concentration *n* > 7 × 10^20^ cm^–3^ and low resistivity ρ of 3.35 × 10^−4^ Ω·cm, while when using a Ar/O_2_(0.4%) gas mixture as a working gas, the carrier concentration decreased down to 2.7 × 10^20^ cm^−3^, but the Hall mobility µ increased by 50% to a value of 39.2 cm^2^/V·s, which in combination resulted in a not so dramatic increase in resistivity ρ up to 5.5 × 10^−4^ Ω·cm (see the Supplementary Materials (SM), Figure S1). Further in the text when referring to donor-rich thin-film IZO deposited under an Ar gas environment the abbreviation *n*-IZO will be used. For another type of high-mobility thin-film IZO the abbreviation µ-IZO will be used. In addition, the IZO films deposited in the presence of O_2_ were characterized by a significantly higher average transmittance compared to the *n*-IZO film in both the visible and near infrared spectral regions despite significant absorption in the short-wavelength region (see SM, Figure S2).

Based on these results, we formed multilayered structures consisting of interleaved *n*-IZO and μ-IZO unit thin layers.

A home-made RF magnetron sputtering setup was used to deposit the *N* × [*n*-ZnO/µ-ZnO] multilayers (MLs) at working gas pressure of 0.5 Pa from 2-inch IZO disk targets with In_2_O_3_/ZnO weight ratio of 9:1 onto glass substrates heated to 100 °C. A detailed description of the experimental setup is given in [29,32]. Switching between the *n*-IZO and µ-IZO deposition modes was carried out by controlled periodic change in the composition of the working gas from pure Ar to an Ar/O_2_(0.4%) gas mixture.

**Figure 1:**
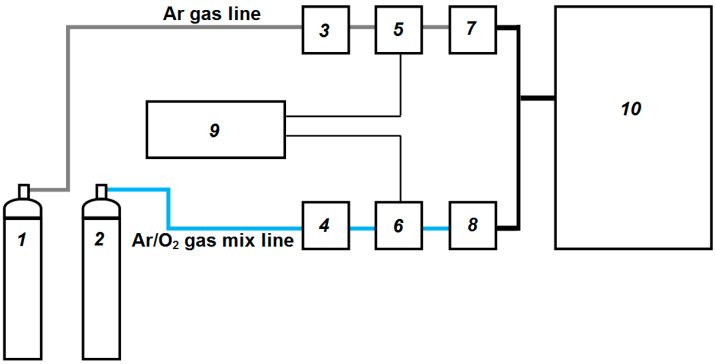
Functional scheme of the two-channel gas supply system. Explanation of the numbers is in the text below.

For a precise programmable change in the composition of the working gas during the ML deposition, the sputtering setup was equipped with a two-channel gas supply system, the functional scheme of which is shown in Figure 1.

Ar gas and Ar/O_2_ gas mix, each in its own line on the way from the gas cylinders (1, 2) to the vacuum chamber (10), sequentially pass through the gas mass flow controllers (GMFC) (3, 4), shut-off valve (5, 6) and the vacuum needle valve (VNV) (7, 8). Initially, for each gas line with its shut-off valve open (with closed valves on the second line), the required working pressure of 0.5 Pa in the vacuum chamber at a flow rate through the GMFC of 20 sccm was achieved by fine tuning the degree of opening of the VNV. Further, during sputtering deposition the supply of Ar or Ar/O_2_ gas mixture to the vacuum chamber was carried out by applying a control signal from the automatic control unit (9) to the shut-off valves (5, 6) at preset degrees of opening of the GMFC and VNV. To reduce the inertia of the gas supply system, all the gas pipeline sections between shut-off valves (5, 6) and the vacuum chamber (10) were made of thin pipes of minimal length.

The total deposition time for each of the *N* × [*n*-ZnO/µ-ZnO] MLs was 200 min. Taking into account that the growth rates of *n*-ZnO and µ-ZnO films were the same (~2 nm/min), the thickness of all MLs was equal to ~400 nm, regardless of the program for changing the composition of the working gas.

The sheet resistance *R_S_* of the IZO-based MLs was measured by using a four-point technique (IUS-3, Moscow, Russia), while electrical transport properties were characterized from Hall effect measurements at room temperature by using the van der Pauw geometry [33]. The optical transmittance of the IZO-based MLs coated on glass substrates was recorded by a dual-beam optical spectrophotometer (Shimadzu UV-3600, Tokyo, Japan) in the wavelength range of 300–1250 nm.

TE quality of the deposited MLs was evaluated by using Haacke’s figure of merit (FOM), defined as FOM = *T*^10^/*R_S_*, where *T* is the average optical transmittance of the ML in the visible range of 400–700 nm [34].

The 400 nm total thickness of all the deposited MLs was confirmed by both scanning electron microscopic measurements (SEM Leo-1450, Karl Zeiss, Jena, Germany) and optical transmission spectra measurements. The amorphous structure of the deposited IZO was verified by means of X-ray diffractometry in the Bragg–Brentano focusing geometry (XRD X’PERT PRO MPD, PANalytical, Malvern, UK).

## 3. Results and Discussion

A systematic investigation was conducted firstly varying the [n-ZnO/µ-ZnO] unit pair thickness in 400 nm thick *N* × [*n*-IZO/µ-IZO] MLs. Moreover, in this experiment, the durations of IZO sputtering in Ar gas and Ar/O_2_ gas mix were the same, so the following equality was valid for thicknesses of the *n*-IZO and µ-IZO unit layers forming MLs:

(1) $$d_{n-\mathrm{IZO}}=d_{\mu -\mathrm{IZO}}=\frac{D}{2\times N}=\frac{400\;\mathrm{nm}}{2\times N}\;,$$

where *d_n_*_–IZO_ and *d*_µ–IZO_ are the thicknesses of *n*-IZO and µ-IZO unit layers, respectively, *D* is the total thickness of the ML, *N* is the number of the (n-ZnO/µ-ZnO) unit pairs.

**Figure 2:**
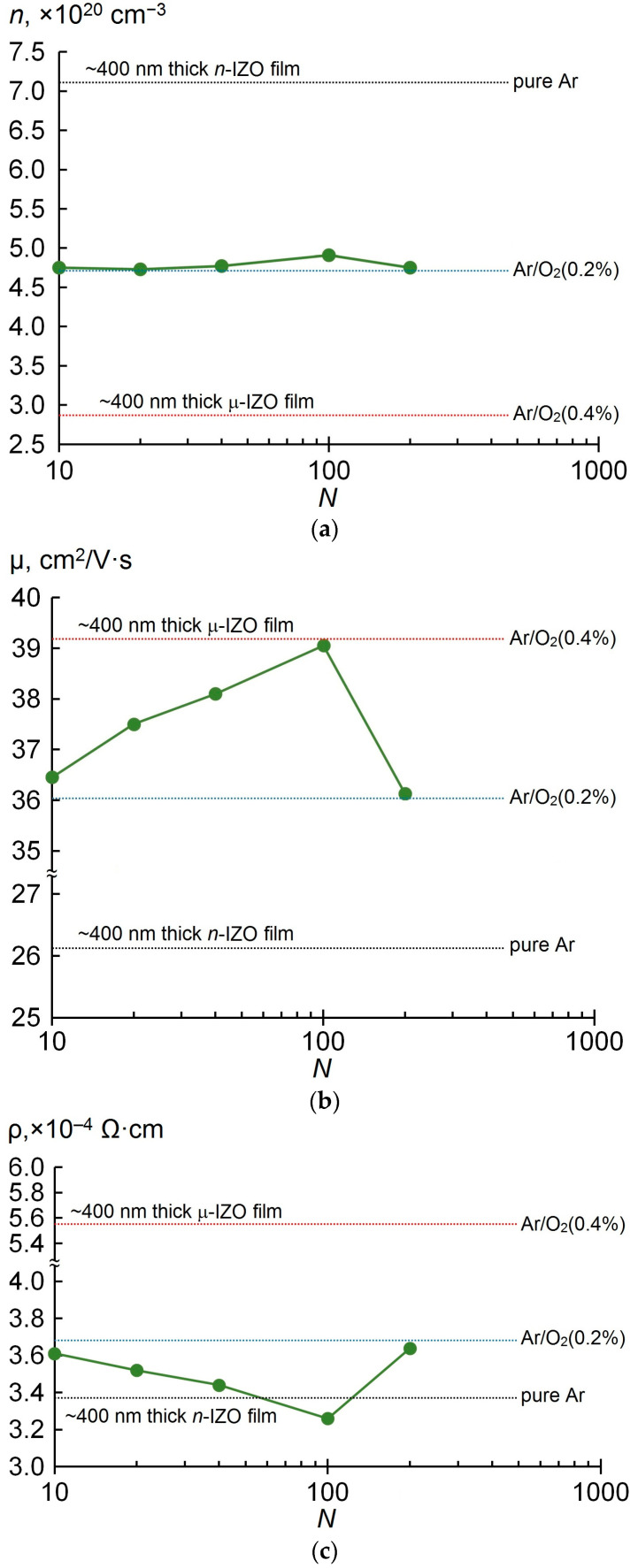
Carrier concentration *n* (**a**), Hall mobility μ (**b**) and resistivity ρ (**c**) of *N* × [*n*-IZO/µ-IZO] MLs as a function of *N*.

Five *N* × [*n*-ZnO/µ-ZnO] MLs with *N* of 200, 100, 40, 20 and 10 were deposited, in which *d_n_*_–ZnO_ and d_µ–ZnO_ were 1, 2, 5, 10 and 20 nm. Figure 2 shows the measured values of Hall mobility μ, carrier concentration *n* and resistivity ρ as a function of the number of unit pairs *N* in the *N* × [*n*-IZO/µ-IZO] MLs. For comparison, dashed lines in Figure 2 also indicate the levels of ρ, μ and *n* values for ~400 nm thick single-layer homogeneous IZO films, obtained under stationary conditions of the working gas supply, at an oxygen content in the working gas of 0 (dashed black), 0.2 (dashed blue) and 0.4% (dashed red) (in accordance with the data illustrated in Figure S2 of the SM).

It can be seen that at *d_n_*_-IZO_ = d_µ-IZO_ in the *N* × [*n*-IZO/µ-IZO] MLs the carrier concentration *n* depends little on *N* and is of the order of ~4.7 × 10^20^ cm^−3^, corresponding to the one for the homogeneous IZO film deposited in the Ar/O_2_(0.2%) working gas (Figure 2a).

At the same time, the Hall mobility μ exhibits a significant dependence on the number of unit pairs *N*. First, it should be noted that the Hall mobility μ in the deposited MLs was noticeably higher than in the homogeneous *n*-IZO film obtained in pure Ar. With an increase in *N* from 10 to 100, i.e., with a decrease in the thickness of [*n*-IZO/µ-IZO] unit pairs from 40 to 4 nm, μ increased to 39 cm^2^/V·s, tending to the value observed in the homogeneous µ-IZO film deposited in the Ar/O_2_(0.4%) gas mix. A further increase in *N* to 200 results in an abrupt drop in μ to the value of 36 cm^2^/V·s, which corresponds to the value for the homogeneous IZO film deposited in the Ar/O_2_(0.2%) gas mix.

The increase in μ with increasing *N* from 10 to 100 results in the fact that the resistivity ρ of the *N* × [*n*-IZO/µ-IZO] MLs has an opposite tendency to fall for this range of *N* (Figure 2c). The 100 × [*n*-IZO/µ-IZO] ML demonstrates resistivity ρ of 3.25 × 10^−4^ Ω·cm that is slightly lower than the resistivity of the homogeneous *n*-IZO film deposited in pure Ar medium. It is due to the high Hall mobility comparable to the one of the homogeneous μ-IZO film, as well as a rather high carrier concentration, close to the one of the homogeneous IZO film deposited at 0.2% O_2_ content.

Thus, in our investigation of the electrical characteristics of the 400 nm thick *N* × [*n*-IZO/µ-IZO] MLs as a function of the number of pairs *N* for the case when $d_{n-\mathrm{IZO}}=d_{\mu -\mathrm{IZO}}$, it was found that the carriers’ Hall mobility is much more sensitive to the thickness of *n*-IZO and µ-IZO unit layers than the carrier concentration.

The insensitivity of *n* to the *N* change is because $d_{n-\mathrm{IZO}}=d_{\mu -\mathrm{IZO}}$. Indeed, the total carrier concentration *n* in the ML consisting of *n*-IZO layers (with a high carrier concentration level) and μ-IZO layers (with lower carrier concentration) is determined by the following expression:

(2) $$n=n_{n-\mathrm{IZO}}\times N\frac{d_{n-\mathrm{IZO}}}{400\;\mathrm{nm}}+n_{\mu -\mathrm{IZO}}\times N\frac{d_{\mu -\mathrm{IZO}}}{400\;\mathrm{nm}}\;,$$

where *n_n_*_–IZO_ and *n*_µ–IZO_ are the carrier concentrations of *n*-IZO and µ-IZO unit layers, respectively.

Taking into account equality (1), expression (2) takes a simpler form, confirming that the total concentration in MLs with equal unit layer thicknesses does not depend on *N*. Therefore, *n* is the arithmetic mean of *n_n_*_-IZO_ and *n*_µ-IZO_:

(3) $$n=\frac{1}{2}(n_{n-\mathrm{IZO}}+n_{\mu -\mathrm{IZO}})\;,$$

At the same time, the observed features of μ dependent on *N* can be explained by the fact that some of the free carriers from the interfaces of the donor-rich *n*-IZO unit layers move to the adjacent regions of the high-mobility μ-IZO layers [21,22,23,24]. In this case, with a decrease in the thickness of unit layers up to 2 nm (*N* = 100), the fraction of overleaped free electrons from *n*-IZO to μ-IZO increases. As a consequence, the contribution to the ionized impurity scattering limitation in carrier mobility should decrease. The observed decrease in Hall mobility in the 200 × [*n*-IZO/µ-IZO] ML, consisting of 400 IZO unit layers with a predicted thickness of 1 nm, can be explained by the inertia of the two-channel gas supply system. In fact, this ML differs little from a homogeneous IZO film deposited in the Ar/O_2_(0.2%) gas mixture.

**Figure 3:**
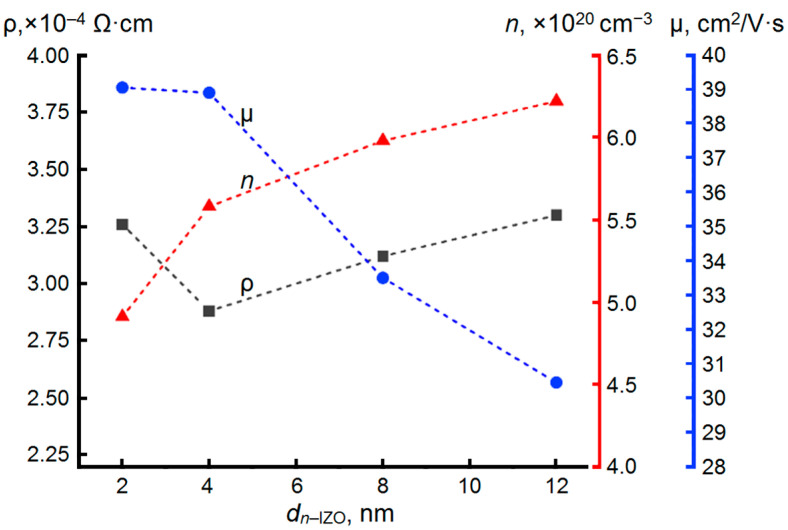
Carrier concentration *n*, Hall mobility μ and resistivity ρ of *N* × [*n*-IZO/µ-IZO] MLs with *d*_µ–IZO_ = 2 nm as a function of *d_n_*_–IZO_.

Further, the influence of the thickness of the *n*-IZO unit layer, enriched with oxygen vacancies, at a constant thickness of a highly mobile μ-IZO unit layer (*d*_µ–IZO_ = 2 nm) on the electrical characteristics of MLs composed of these layers was additionally studied. Obviously, in the ML the total concentration of charge carriers *n* should increase with an increase in *d_n_*_–IZO_ at constant *d*_µ–IZO_ = 2 nm. However, this reduces the number of pairs *N* in the ML (as well as number of *n*-IZO/µ-IZO interfaces) too. Indeed, with an increase in *d_n_*_–IZO_, there are tendencies of an increase in *n* and a decrease in µ (Figure 3).

However, it should be noted that when *d_n_*_-IZO_ increases from 2 to 4 nm, the concentration *n* increases by almost 10% (from 4.90 × 10^20^ to 5.55 × 10^20^ cm^−3^), while the Hall mobility µ decreases by less than 1% (from 39 to 38.7 cm^2^/V·s). Therefore, for the case of the ML in which *d_n_*_-IZO_ = 2 × *d*_µ-IZO_ = 4 nm, a minimum of resistivity ρ of 2.9 × 10^–4^ Ω·cm is observed. A further increase in *d_n_*_-IZO_ to 12 nm leads to a sharper decrease in µ (by about 30%) against the background of a gradually rising concentration *n*, which generally results in a trend towards an increase in ρ up to 3.3 × 10^–4^ Ω·cm.

Thus, the most interesting from the point of view of the best electrical performance are 400 nm thick MLs formed from alternating 2 nm thick µ-IZO unit layers and *n*-IZO layers with thickness values of 2 and 4 nm among the deposited MLs. The first ML based on a repeating [*n*-IZO_2 nm_/µ-IZO_2 nm_] pair is characterized by the highest value of Hall mobility μ of 39 cm^2^/V·s with ρ = 3.2 × 10^–4^ Ω·cm. The second optimized ML structure is many stacked [*n*-IZO_4 nm_/µ-IZO_2 nm_] pairs, which demonstrates the lowest resistivity ρ = 2.90 × 10^–4^ Ω·cm due to the high total concentration (*n* = 5.55 × 10^20^ cm^–3^) and a just a little less Hall mobility (µ = 38.7 cm^2^/V·s) than that in the former ML structure.

It should also be noted that both ML types exhibit Hall mobility values comparable to that observed in a homogeneous highly mobile µ-IZO film obtained by sputtering in an An/O_2_(0.4%) gas mix medium. At the same time, the MLs are characterized by higher carrier concentration than homogeneous µ-IZO, which is due to the presence of donor-rich *n*-IZO ultrathin layers in the multilayer structure.

**Figure 4:**
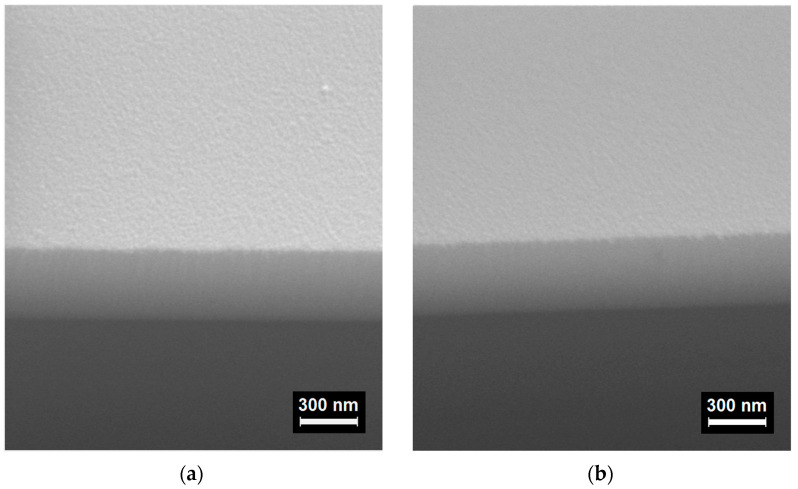
SEM morphology images of the 400 nm thick MLs: *N* × [*n*-IZO_2 nm_/μ-IZO_2 nm_] (**a**); *N* × [*n*-IZO_4 nm_/μ-IZO_2 nm_] (**b**).

Figure 4 shows micrographs of the 400 nm thick MLs with optimized thicknesses of unit layers. It can be seen that their morphology differs little both from each other and from the morphology of homogeneous films presented in Figure S3 of the SM. This is because the smooth morphology of the low-temperature IZO films does not depend on the presence or absence of O_2_ in the working gas [25,26,29]. A characteristic very smooth surface of both IZO-based MLs, as well as the homogeneous IZO films, is typical for the case of a closely packed multicomponent amorphous film structure containing oxide components with weak mutual solubility [25,35]. The surface morphology of two ML samples was additionally tested by atomic force microscopy (AFM Ntegra Prima, Moscow, Russia), as shown in Figure S4 of the SM. Both MLs feature a slight roughness, and the root-mean-squared values are 0.486 nm for *N* × [*n*-IZO_4 nm_/μ-IZO_2 nm_] and 0.509 nm for *N* × [*n*-IZO_2 nm_/μ-IZO_2 nm_]. Thus, the microscope studies confirm the improved surface smoothness of the MLs, characteristic for amorphous IZO thin-film materials deposited at low substrate temperatures [25,36].

**Figure 5:**
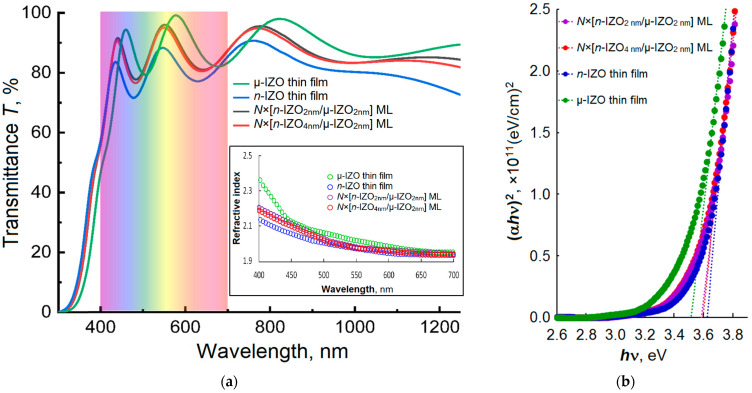
Optical transmittance spectra (**a**) and the plots of (α*h*ν)^2^ = f(*h*ν) (**b**) for the *n*-IZO and µ-IZO homogeneous films and the MLs with optimized thicknesses of unit layers. The inset shows the refractive index dispersion in the visible range.

The optical transmittance spectra of the optimized 400 nm thick IZO-based MLs (*N* × [*n*-IZO_2 nm_/μ-IZO_2 nm_] and *N* × [*n*-IZO_4 nm_/μ-IZO_2 nm_]) are shown in Figure 5a. For comparison, Figure 5 shows the spectra of the homogeneous IZO films of the same thickness. The optical transmission level in the visible range (400–750 nm) of the MLs is less than that of the homogeneous µ-IZO film, but higher than that of the *n*-IZO film. The values of the average optical transmittance $\bar{T}\;$of the IZO MLs with optimized architecture calculated for visible range of 400–700 nm are presented in Table 1.

In the MLs’ spectra, a significant short-wavelength shift of the fundamental absorption edge relative to the spectrum of the homogeneous µ-IZO film is observed, which leads to a strong increase in transmittance in the short-wavelength region of the visible range (in the neighborhood of 400 nm), similar to that observed in the *n*-IZO film. That can be attributed to the well-known Burstein–Moss shift due to the higher concentration *n* in both types of MLs compared to that in the homogeneous μ-IZO film [37]. The direct bandgap optical absorption model closely describes the absorption edge of amorphous IZO, as well as several other amorphous and crystalline TCOs [36,38,39]. The values of *E_g_* were obtained by extrapolating the linear part of the plots of (α*h*ν)^2^ vs. *h*ν to the zero absorption level (α = 0), as shown in Figure 5b. The MLs’ *E*_g_ values determined are 3.58 eV for the *N* × [*n*-IZO_2 nm_/μ-IZO_2 nm_] sample and 3.60 eV for the *N* × [*n*-IZO_4 nm_/μ-IZO_2 nm_]. These values are less than the *E_g_* value of the homogeneous *n*-IZO film (3.63 eV), but greater than *E_g_* = 3.51 eV measured for the homogeneous μ-IZO film. The observed *E_g_* variation confirms the results of the Hall effect measurements. It can be ascribed to the change in carrier concentration in the IZO homogeneous films and MLs depending on the composition and switching mode of the working gas.

The refractive index dispersion, determined using the pointwise unconstrained minimization approach during the fitting of theoretical transmission spectra with experimental ones [40], shows an expected monotonically decrease with growing wavelength λ for both IZO MLs, as well as n-IZO and m-IZO homogeneous films, as shown in the Figure 5 inset. The obtained values of refractive index in the middle of the visible optical range (at λ = 550 nm) are between 2.02 and 1.97, closely in agreement with values reported for amorphous IZO [2,26,41,42].

Table 1 presents the results of the evaluated TE quality of the homogeneous *n*-IZO and µ-IZO, as well as the IZO MLs with optimized structure, by using the generally accepted Haacke’s FOM. It can be seen that the FOM of the optimized MLs is almost twice that of homogeneous IZO films of the same thickness. High TE quality of the MLs is because they simultaneously demonstrate a conductivity behavior similar to the homogeneous *n*-IZO film deposited in pure Ar and a high transparency characteristic in the visible range, characteristic for homogeneous µ-IZO films deposited in an Ar/O_2_ gas mix.

## 4. Conclusions

Programmable periodic changes in the O_2_ content in the working gas during RF magnetron sputtering of IZO targets were used to form the transparent conducting *N* × [*n*-IZO/µ-IZO] multilayer structures. The effect of thicknesses of donor-rich *n*-IZO and high-mobility μ-IZO unit layers on the electrical properties of the multilayers was investigated. It was found that the electrical characteristics of the multilayers are sensitive to the thicknesses of *n*-IZO and μ-IZO unit layers. The thickness ranges providing highest mobilities combined with low resistivity were 2 nm for the μ-IZO and 2–4 nm for the n-IZO. Mobility as high as 39 cm^2^/Vs was achieved for the *N* × [*n*-IZO_2 nm_/µ-IZO_2 nm_] multilayer with a free carrier concentration of ~5 × 10^20^ cm^−3^ and a resistivity of 3.25 × 10^−4^ Ω·cm. At the same time, with an increase in the thickness of the *n*-IZO unit layer up to 4 nm, for a comparable mobility in the *N* × [*n*-IZO_2 nm_/µ-IZO_2 nm_] multilayer, the resistivity was further reduced to 2.9 × 10^−4^ Ω·cm due to increasing carrier concentration up to 5.5 × 10^20^ cm^−3^. As shown by the AFM and SEM measurements, improved surface smoothness, which is typical for amorphous IZO, is also preserved in the electrically optimized multilayers. The combination of donor-rich and high-mobility IZO ultrathin layers in the multilayer structures also improves the optical transmittance towards values inherent in high-mobility μ-IZO films and widens the bandgap towards values characteristic of donor-rich *n*-IZO films.

Thus, magnetron sputtering deposition of the multilayer structures based on multiple alternations of the high-concentration IZO and the high-mobility IZO unit layers, achieved by working gas composition cycling during sputtering, can be an effective tool for improving the TE quality of low-temperature IZO-based TCO. The TE quality improvement for the IZO multilayers is apparently associated with some mitigation of the limitations of ionized impurity scattering in them due to the spatial separation of areas with high carrier concentrations and areas with improved carrier transport behavior.

## Figures and Tables

**Table 1:** TE quality of IZO-based TCO thin-film structures.

Type of IZO Thin-Film Structure	Sheet Resistance *R*, Ω/sq	Average Transmittance ${\bar{T}}_{400–700\;\mathrm{nm}}$, %	FOM, Ω^–1^
Homogeneous *n*-IZO film	8.38	79.1	1.14 × 10^–2^
Homogeneous µ-IZO film	13.88	84.2	1.29 × 10^–2^
*N* × [*n*-IZO_2 nm_/μ-IZO_2 nm_] ML	8.13	84.5	2.28 × 10^–2^
*N* × [*n*-IZO_4 nm_/μ-IZO_2 nm_] ML	7.25	83.8	2.36 × 10^–2^

## Data Availability

Not applicable.

## Supplementary Materials

The following supporting information can be downloaded at: https://www.mdpi.com/article/10.3390/ma16103740/s1, Figure S1: Electrical properties of the IZO thin films deposited at 100 °C as a function of the O_2_ content in the working gas; Figure S2: Optical transmittance of the IZO films deposited at substrate temperatures of 100 °C with varying O_2_ contents; Figure S3: SEM morphology images of the IZO films deposited under various O_2_ contents: a—pure Ar; b—Ar/O_2_(0.4%); c—Ar/O_2_(0.5%); Figure S4: The 2D AFM images of the surface of the *N* × [*n*-IZO_2 nm_/μ-IZO_2 nm_] ML (a) and *N* × [*n*-IZO_4 nm_/μ-IZO_2 nm_] ML (b).
