# Supplementary material for: Transparent Conducting Amorphous IZO Thin Films: An Approach to Improve the Transparent Electrode Quality
Source: Materials (Basel). 2023 May 15;16(10):3740. doi: 10.3390/ma16103740 (PMC10223404; doi:10.3390/ma16103740)
Supplement: Supplementary file 1 [file materials-16-03740-s001.zip › materials-2374001-supplementary.pdf]

materials

Supplementary Materials to the article

Transparent Conducting Amorphous IZO Thin Films: An Approach to Improve the Transparent Electrode Quality

Akhmed K. Akhmedov [1], Aslan Kh. Abduev [2,3], Eldar K. Murliev [1], Victor V. Belyaev [2,3] and Abil Sh. Asvarov [1,*]

[1] Institute of Physics, Dagestan Research Center of Russian Academy Sciences, Yaragskogo Str., 94, 367015 Makhachkala, Russia
[2] Faculty of Physics and Mathematics, State University of Education, Very Voloshinoi Str. 24, 141014 Mytishchi, Russia
[3] RUDN University, 6, Miklukho-Maklay Str., 117898 Moscow, Russia
* Correspondence: abil-as@list.ru

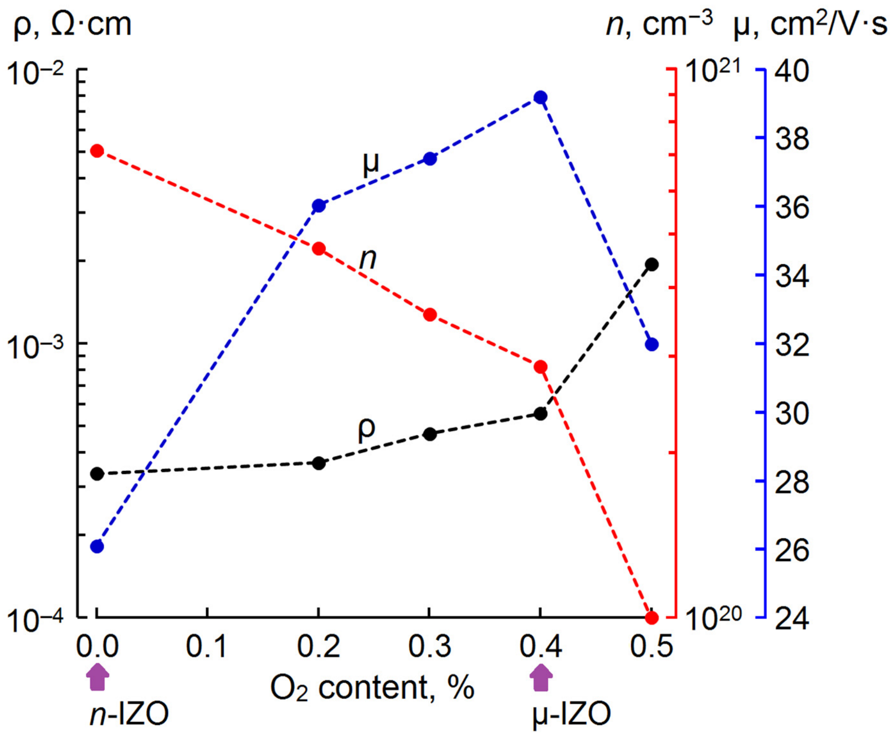

Figure S1. Electrical properties of the IZO thin films deposited at 100°C as a function of the O_2 content in the working gas.

Violet arrows mark the values of O_2 content at which *n*-IZO and μ-IZO unit layers of IZO-based multilayer structures were formed, respectively.

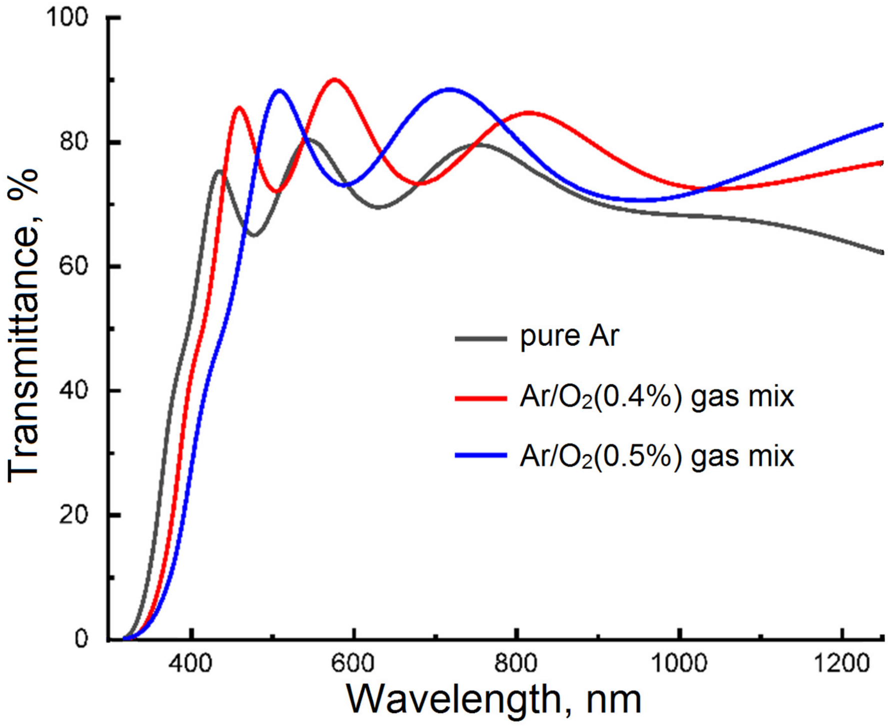

Figure S2. Optical transmittance of the IZO films deposited at substrate temperatures of 100 °C with varying O_2 contents.

It can be seen that, as the O_2 content decreases, the absorption edge of IZO/glass samples shifts to shorter wavelengths, but the overall level of optical transmission in the "transparency window" of IZO TCO samples decreases.

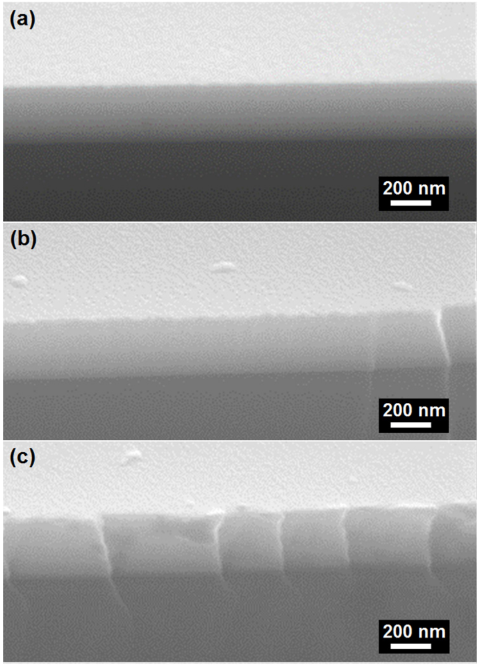

Figure S3. SEM morphology images of the IZO films deposited under various O_2 content: a – pure Ar; b – Ar/O_2(0.4%); c - Ar/O_2(0.5%).

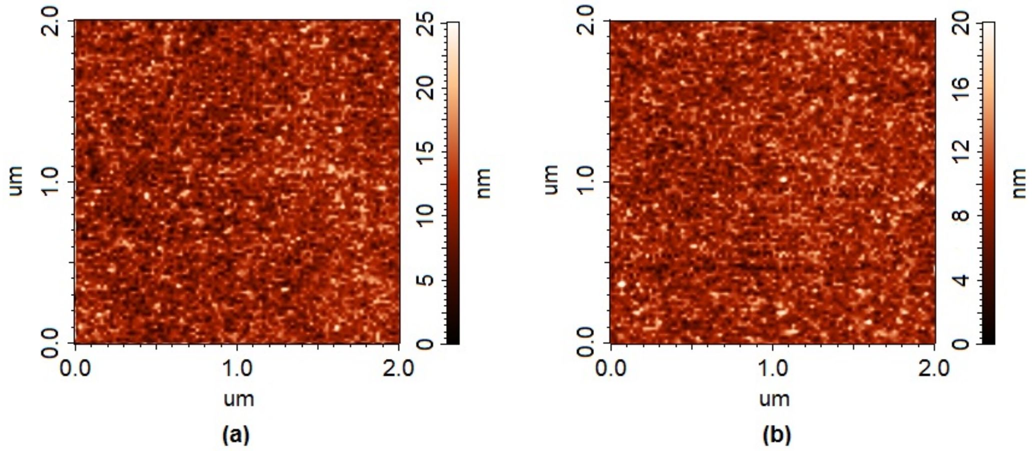

Figure S4. 2D AFM images of the surface of the N×[n-IZO$_{2\,nm}$/μ-IZO$_{2\,nm}$] ML (a) and N×[n-IZO$_{4\,nm}$/μ-IZO$_{2\,nm}$] ML (b).
